# 2317. The Impact of Universal versus Targeted Symptomatic COVID-19 Arrival Testing in Basic Military Trainees

**DOI:** 10.1093/ofid/ofad500.1939

**Published:** 2023-11-27

**Authors:** Marquise Westbrook, James Aden, John Kieffer, Erin Winkler, Theresa Casey, Dianne Frankel, Angela Osuna, John Kiley, Heather Yun, Joseph Marcus

**Affiliations:** Brooke Army Medical Center, San Antonio, Texas; BAMC, San Antonio, Texas; BAMC, San Antonio, Texas; BAMC, San Antonio, Texas; BAMC, San Antonio, Texas; Trainee Health Surveillance, San Antonio, Texas; BAMC, San Antonio, Texas; BAMC, San Antonio, Texas; Brooke Army Medical Center, San Antonio, Texas; Brooke Army Medical Center, San Antonio, Texas

## Abstract

**Background:**

The ideal strategy for COVID-19 testing upon arrival to a congregate setting is unknown. Basic Military Training (BMT) is 7.5 weeks long and service members live in groups of 35-50 individuals in open bay dorms for the entirety of their training. The United States Air Force BMT transitioned from universal arrival antigen testing to targeted symptomatic antigen testing on October 26, 2021. This study evaluates COVID-19 positivity rates in the first two weeks of BMT when trainees underwent universal testing vs targeted arrival testing.

**Methods:**

All COVID positive trainees were identified from August 1, 2021-December 15, 2021. The risks of an individual as well as a training group developing a case on arrival or in the first fourteen days of training were compared between those who underwent universal arrival antigen testing (August 1-October 26) vs. targeted testing (October 26-December 15). A case cluster was considered if there were > 5 cases in one training group. Nominal variables were compared by Chi-squared analysis and continuous variables were compared by Mann-Whitney U test.

**Results:**

During the study period, 13,384 trainees began BMT, and there were 690 (5.2%) cases of COVID-19 in the first two weeks of training. Regardless of testing strategy, there was no difference in trainees testing positive on arrival (1.4% vs. 1.3%, p=0.68). However, more trainees in the universal screening group tested positive on day 2-14 of training (3.9% vs. 2.8%, p= 0.002). When evaluating training groups, universally tested training groups did not have lower rates of developing a case cluster (33/271 (12.1%) vs. 6/104 (5.8%), p=0.09) as compared to targeted symptomatic testing, respectively.

**Conclusion:**

In this large cohort during the COVID-19 Delta wave, targeted antigen testing was not associated with increased secondary cases of COVID-19 in a congregate setting. This study supports the feasibility of the use of targeted symptomatic testing for individuals entering congregate settings.

**Disclosures:**

**All Authors**: No reported disclosures

